# Limited solvation of an electron donating tryptophan stabilizes a photoinduced charge-separated state in plant (6–4) photolyase

**DOI:** 10.1038/s41598-022-08928-0

**Published:** 2022-03-24

**Authors:** Yuhei Hosokawa, Pavel Müller, Hirotaka Kitoh-Nishioka, Shigenori Iwai, Junpei Yamamoto

**Affiliations:** 1 Graduate School of Engineering Science, Osaka University, 1-3 Machikaneyama, Toyonaka, Osaka 560-8531 Japan; 2 Université Paris-Saclay, CEA, CNRS, Institute for Integrative Biology of the Cell (I2BC), 91198 Gif-sur-Yvette, France; 3 Graduate School of System Informatics, Kobe University, 1-1 Rokkodai, Nada-ku, Kobe, 657-8501 Japan

**Keywords:** Biophysical chemistry, Biophysics

## Abstract

(6–4) Photolyases ((6–4) PLs) are ubiquitous photoenzymes that use the energy of sunlight to catalyze the repair of carcinogenic UV-induced DNA lesions, pyrimidine(6–4)pyrimidone photoproducts. To repair DNA, (6–4) PLs must first undergo so-called photoactivation, in which their excited flavin adenine dinucleotide (FAD) cofactor is reduced in one or two steps to catalytically active FADH^−^ via a chain of three or four conserved tryptophan residues, transiently forming FAD^•−^/FADH^−^ ⋯ TrpH^•+^ pairs separated by distances of 15 to 20 Å. Photolyases and related photoreceptors cryptochromes use a plethora of tricks to prevent charge recombination of photoinduced donor–acceptor pairs, such as chain branching and elongation, rapid deprotonation of TrpH^•+^ or protonation of FAD^•−^. Here, we address *Arabidopsis thaliana* (6–4) PL (*At*64) photoactivation by combining molecular biology, in vivo survival assays, static and time-resolved spectroscopy and computational methods. We conclude that *At*64 photoactivation is astonishingly efficient compared to related proteins—due to two factors: exceptionally low losses of photoinduced radical pairs through ultrafast recombination and prevention of solvent access to the terminal Trp_3_H^•+^, which significantly extends its lifetime. We propose that a highly conserved histidine residue adjacent to the 3rd Trp plays a key role in Trp_3_H^•+^ stabilization.

## Introduction

Photolyases and cryptochromes constitute a superfamily (PCSf) of ubiquitous structurally related photoactive flavoproteins^1,2^. The evolutionarily older photolyases (PLs) have specialized in the photoenzymatic repair of major UV-induced lesions: cyclobutane pyrimidine dimers (CPDs; specifically repaired by CPD PLs) and pyrimidine(6–4)pyrimidone photoproducts ((6–4)PPs; specifically repaired by (6–4)PLs). Cryptochromes (CRYs), which branched off from photolyases, have gradually lost their ability to repair DNA, but have progressively acquired new physiological functions as blue light receptors driving photomorphogenesis in plants or entraining the circadian clock in both plants and animals^2^. Numerous theoretical and experimental works suggest that animal cryptochromes, which group together with animal (6–4) photolyases, are also responsible for the ability of migratory birds and other animals to sense the Earth’s magnetic field and use it for orientation^3,4^.

PCSf proteins undergo two distinct kinds of photoreactions: *photorepair* and *photoactivation*. In photorepair, which is unique to photolyases and a few rare ‘dual’ proteins (capable of acting as both DNA repair enzymes and photoreceptors^5–7^), the photoexcited fully reduced flavin (*FADH^−^) transfers an electron to the DNA lesion fixed in a nearby specific binding pocket. This electron transfer (ET) triggers bond rearrangement within the lesion, which, upon electron return to the intrinsically semi-oxidized (or semi-reduced) FADH^•^, ultimately leads to restoration of two intact bases and thus to DNA repair.

**Figure 1:**
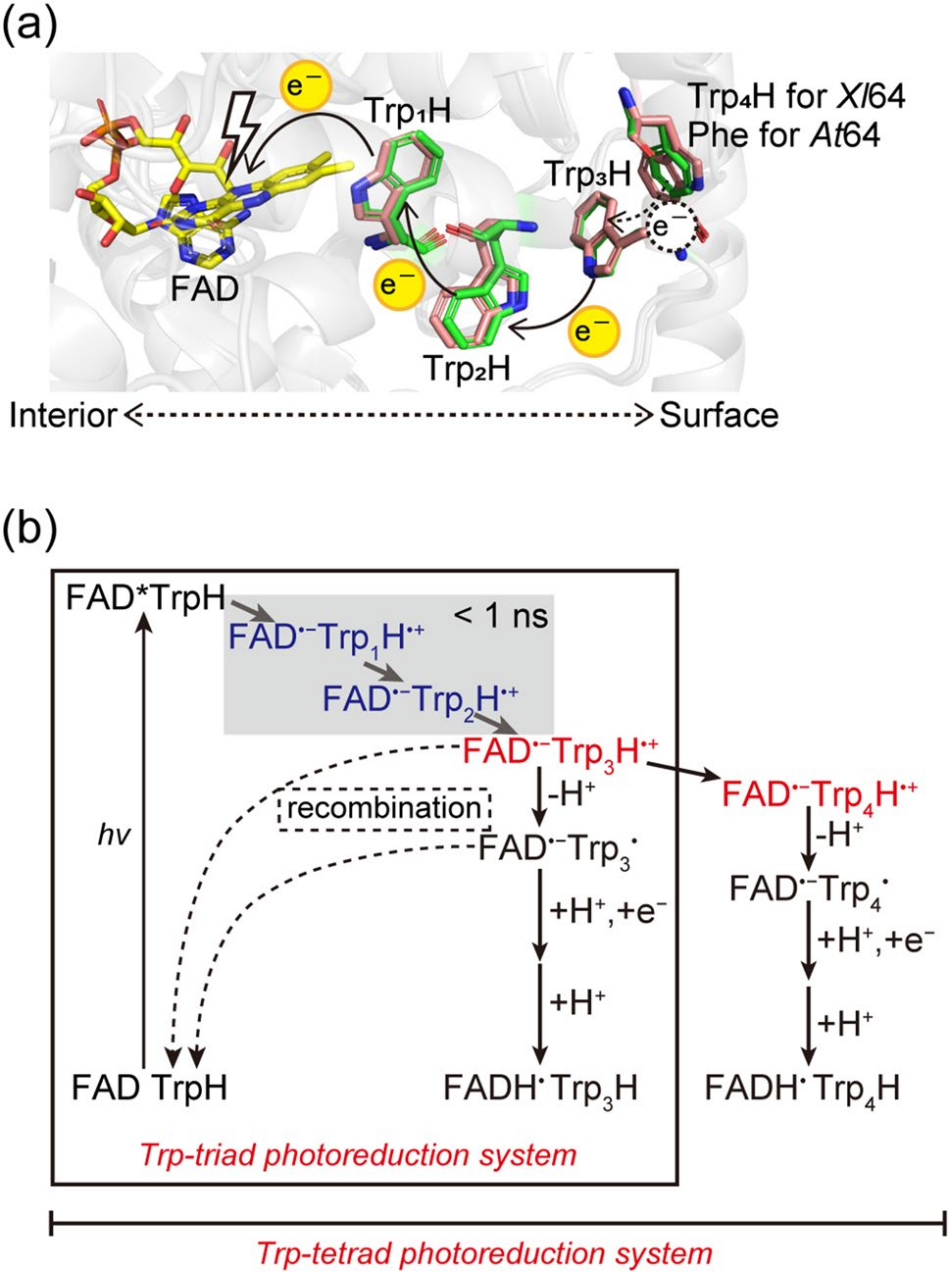
Scheme of FAD photoreduction in PCSf proteins. (**a**) The crystal structure of *At*64 (PDB entry 3FY4)^19^ is superimposed onto a homology model structure of *Xl*64 using the crystal structure of (6–4) PL from *Drosophila melanogaster* (6–4) PL (PDB entry 3CVU)^20^ as template. FAD is shown in yellow, electron-transferring tryptophans and redox-inactive phenylalanine in *At*64 and *Xl*64 are shown in green and in salmon, respectively. (**b**) Reaction scheme detailing typical photo-induced charge transfer, charge separation and radical stabilization in PCSf proteins with Trp triads and tetrads. TrpH, TrpH^•+^, and Trp^•^ denote the normal (non-oxidized) tryptophan state, the one-electron-oxidized cation radical, and the one-electron-oxidized deprotonated neutral radical, respectively.

Nevertheless, the flavin cofactor in PLs is not always in the catalytically active (fully reduced) form and therefore needs to be activated (reduced). This happens in the latter reaction called photoactivation. Photoactivation, which is common to both PLs and CRYs, is a light-induced reduction of the oxidized (FAD_ox_) or semi-reduced (FADH^•^) flavin chromophore via a chain of electron-transferring aromatic residues, typically three tryptophans. Upon excitation, *FAD_ox_ or *FADH^•^ abstract an electron from a nearby Trp residue^8^ (Trp_1_H), producing a ~ 4 Å charge-separated state (FAD^•−^/FADH^−^ ⋯ Trp_1_H^•+^). Trp_1_H^•+^ subsequently gets an electron from a second Trp residue^9^ (Trp_2_H), and the resulting radical cation Trp_2_H^•+^ in turn acquires an electron from yet another Trp residue near the protein surface^10–12^ (Trp_3_H), as illustrated in Fig. 1a. The successive ET on the sub-nanosecond time scales yields a ~ 15 Å charge-separated state (FAD^•−^/FADH^−^···Trp_3_H^•+^). Finally, deprotonation^13^ of Trp_3_H^•+^ to Trp_3_^•^ and quenching of Trp_3_^•^ by external reducing agents^14^ stabilize the FAD^•−^/FADH^−^ state^15^ (see Fig. 1b detailing the first photoactivation step starting with FAD_ox_ and yielding the semi-reduced FAD^•−^/FADH^•^). While the catalytically active redox form of FAD in DNA repair by PLs is FADH^−^, light signaling by CRYs is believed to be triggered by FAD_ox_ photoreduction to FAD^•−^ (or by a negative charge on a neighboring residue that protonated FAD^•−^ to FADH^•^)^16–18^. Most CRYs hence seem to use only the first photoactivation step.

To ensure efficient photoactivation, PCSf proteins employ a plethora of ingenious tricks stabilizing the light-induced radicals and preventing futile charge recombination: *e.g.*, branching and/or elongation of the ET chain to four or even five residues^21–23^, rapid (~ µs) protonation of FAD^•−^ (unique to plant cryptochromes)^24^, or rapid (sub-ns) deprotonation of the last member of the ET chain. The latter can be achieved by the presence of a proton acceptor next to the terminal member of the ET chain (*e.g.*, a deprotonated aspartic or glutamic acid^25^ or a cluster of structured water molecules communicating with the surrounding buffer^26^) or by using tyrosine as the terminal residue of the chain^25,26^ (while TrpH^•+^ has a p*K*_a_ of ~ 4^27^, TyrH^•+^ has a p*K*a of ~  − 2^28^ and deprotonates immediately in aqueous buffers).

In this study, we focused on the (6–4) photolyase from *Arabidopsis thaliana* (*At*64), which, despite possessing a mere Trp triad, exhibits surprisingly high activity in bacterial cells—comparable to that of *Xenopus laevis* (6–4) PL (*Xl*64)^29^, which contains a Trp tetrad, and the photoactivation of which is known to yield very stable charge separation and unusually long-lived radical pairs^21^. Our results suggest that *At*64 achieves efficient photoactivation by using a different set of tricks than other PCSf proteins: 1) rapid localization of the electron hole on the 3rd Trp (which we infer from an unusually high (> 80%) quantum yield of the FAD^•−^ Trp_3_H^•+^ pairs, implying proportionally low losses of primary and secondary FAD^•−^ TrpH^•+^ pairs by ultrafast recombination), and 2) prevention of solvent access to Trp_3_H^•+^, which extends its lifetime by a factor of ten (compared to a mutant with solvent-exposed 3rd Trp). We propose that a histidine residue adjacent to the 3rd Trp and highly conserved in plant (6–4) PLs plays a key role in stabilization of Trp_3_H^•+^, thus expanding the arsenal of tricks leading to efficient photoactivation of PCSf proteins.

## Results

### Primary and tertiary structures reveal major differences in the environment of Trp_3_H in plant and animal (6–4) PLs

**Figure 2:**
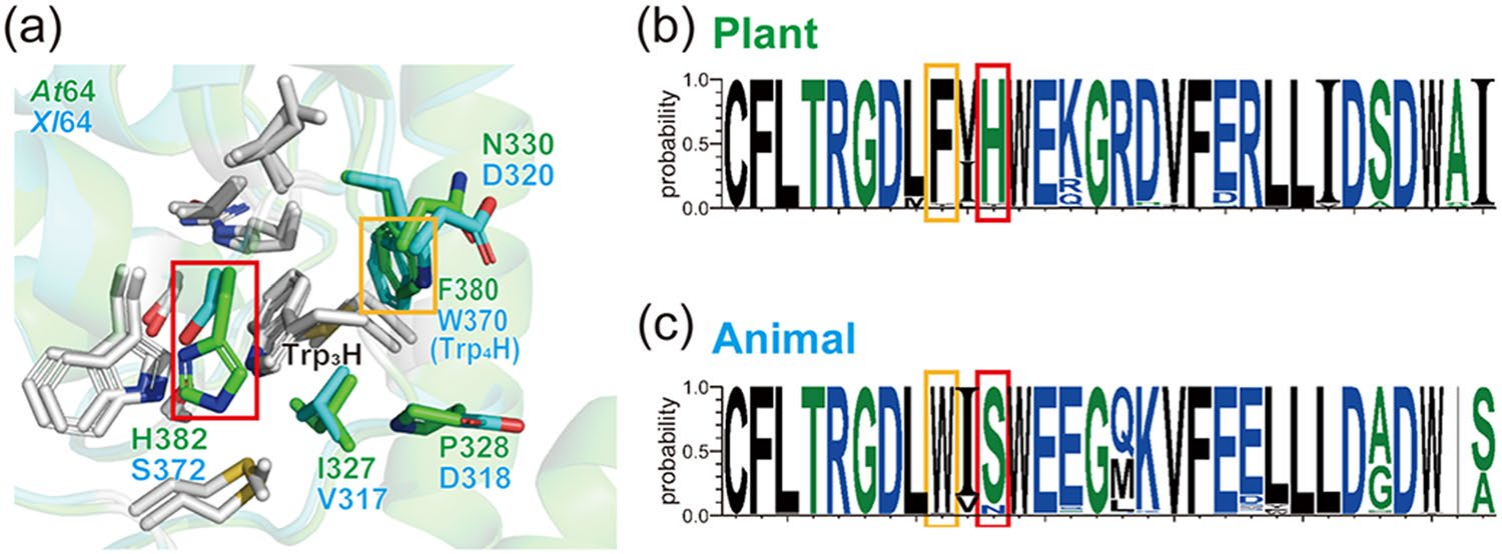
Structure and sequence analyses of plant and animal (6–4) PL orthologues. (**a**) The local three-dimensional structures around Trp_3_H in *At*64 and *Xl*64. Residues conserved in both (6–4) PLs are shown in light grey. Residues unique to *At*64 and *Xl*64 are colored in green and cyan, respectively. (**b**) and (**c**) Primary structural analyses of (**b**) plant and (**c**) animal (6–4) PL orthologues. The phenylalanine inside the yellow box corresponds to F380 in *At*64. The histidine corresponding to H382 in *At*64 is highlighted in red. The electron-transferring Trp_4_H (W370 in *Xl*64) is marked in yellow. The serine inside the red frame corresponds to S372 in *Xl*64.

To address how plant (6–4) PLs are finely adapted to FAD photoreduction via the Trp triad, we assumed that residues around Trp_3_H affect the formation and/or stabilization of the photoinduced FAD^•−^ Trp_3_H^•+^ charge-separated state. To explore the differences in the environment of Trp_3_H in plant and animal (6–4) PLs, the crystal structure of *At*64^19^ (PDB entry 3FY4) was superimposed onto a homology model structure of *Xl*64 generated as previously described^21^ (Fig. 2a). Apart from the phenylalanine residue (Phe380 in *At*64), which is replaced by the fourth tryptophan in animal (6–4) PLs (Trp370 in *Xl*64), the only major difference in the closest neighborhood of Trp_3_H (within 4 Å) is that a histidine residue conserved in plant (6–4) PLs (His382 in *At*64) is substituted by a serine in animal (6–4) PLs (Ser372 in *Xl*64). The remaining residues around Trp_3_H are either conserved (Trp383 and Met318 in *At*64 numbering) or very similar (Ile327 in *At*64 *vs.* Val317 in *Xl*64).

Our sequence analyses revealed that as many as 95% of compared plant (6–4) PL orthologues retain the His residue, and 92% of the animal (6–4) PL orthologues retain the Ser residue (Figs. 2b,c). Interestingly, in no plant (6–4) PL orthologues is the histidine replaced by a serine, suggesting that the histidine residue could play a functional or at least an auxiliary role in the stabilization of Trp_3_H^•+^ and thereby in the photoreduction of plant (6–4) PLs.

### Mutation of His382 to Ser in *At*64 slows down the photoreduction in vitro and impairs the photorepair capability in bacterial cells

**Figure 3:**
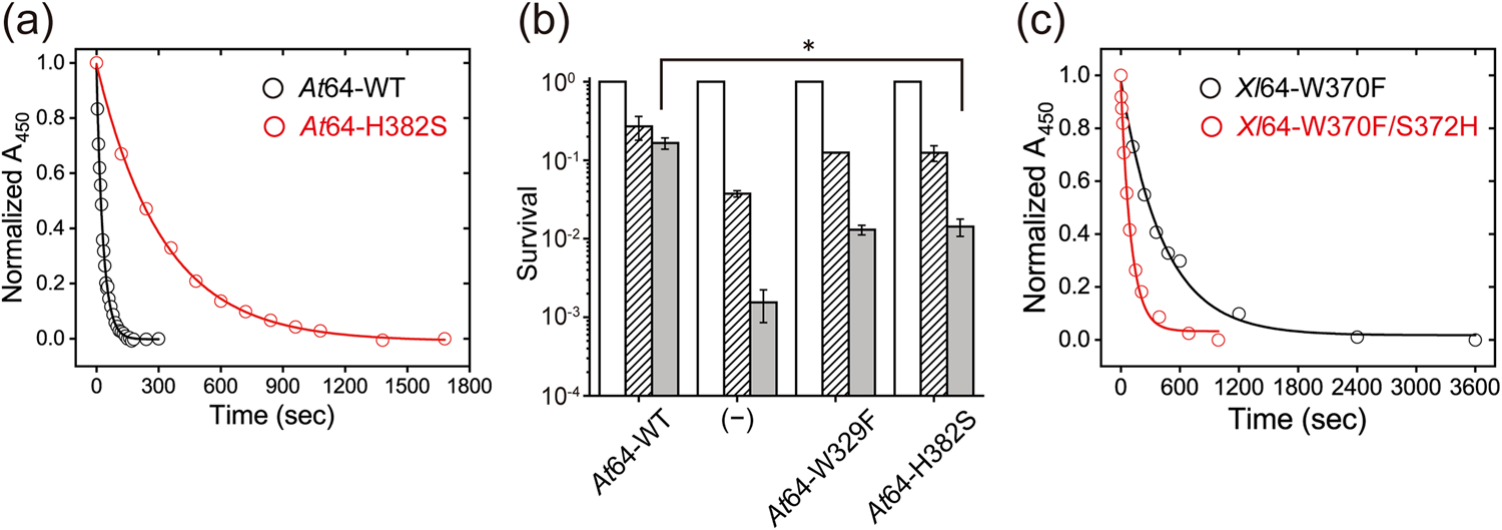
The influence of His → Ser and Ser → His substitutions on the photoreduction of and photorepair by *At*64 and *Xl*64, respectively. (**a**) Comparison of the decay of normalized *A*_450_ reflecting the FAD photoreduction in *At*64-WT and *At*64-H382S. Measured data points (empty circles) are fitted with monoexponential decay functions (solid lines). (**b**) (6–4) PP photorepair activity assay for *At*64 variants in *E. coli* cells. Survivals of the cells transformed with the plasmids encoding WT or mutants of *At*64 or an empty vector (annotated as (-)) upon 0 J m^−2^ (open bar), 0.3 J m^−2^ (shaded bar), and 0.6 J m^−2^ (grey bar) UV irradiation, followed by white light for 30 min. The W329F mutant is the mutant lacking Trp_3_H. The experiments were performed in triplicates (*n* = 3) and the statistical significance was analyzed with a *t*-test, where the significance cutoff value was set to 0.05, and the asterisk indicates the *P* value of 0.0035. (**c**) The decay kinetics of normalized *A*_450_ in the *Xl*64-W370F and *Xl*64-W370F/S372H mutants show that the S372H mutation in *Xl*64-W370F enhances the photoreduction efficiency in *Xl*64 lacking Trp_4_H.

To examine whether the His/Ser difference affects FAD photoreduction via the Trp-triad, we investigated steady-state photoreduction kinetics for the H382S mutant of *At*64 (*At*64-H382S), as previously performed^30^ for the wild-type of *At*64 (*At*64-WT). *At*64-H382S was illuminated by > 430 nm light in the presence of an external reductant under anaerobic conditions and the UV/Vis absorption spectral changes were monitored. Upon light illumination, the absorption band at 450 nm characteristic of FAD_ox_ decayed and the spectra were gradually converted to that of FADH^−^ (via transient accumulation of small amounts of FADH^•^, as indicated by the absorption growth and decay between 500 and 700 nm; Supplementary Fig. 1). Fitting the normalized absorption at 450 nm (*A*_450_) with a monoexponential decay function revealed that FAD photoreduction in *At*64-H382S occurred with a half-life (*t*_1/2_) of 222 ± 4 s, which is about tenfold slower than that in *At*64-WT^30^ (*t*_1/2_ = 21.6 ± 0.5 s) under the same conditions (Fig. 3a). To examine whether the less efficient photoreduction caused by the H382S mutation affects the (6–4)PP photorepair activity of *At*64, UV-sensitive *E. coli* SY32 cells, in which CPD lesions can be repaired due to a rescue plasmid coding *E. coli* CPD PL gene, were transformed with an *At*64-H382S expressing plasmid, in the same way as previously reported^30^. The survival rate was found to be about tenfold lower in H382S than in WT (Fig. 3b). These results indicate that the H382S mutation in *At*64 impairs the (6–4)PP photorepair in bacterial cells by hampering the preceding (and necessary) photoreduction reaction.

In the previous study^29^ on FAD photoreduction in *Xl*64 containing a Trp-tetrad, the mutation of Trp_4_H in *Xl*64 to Phe (*Xl*64-W370F) showed a comparatively slow photoreduction kinetics under the same conditions (Fig. 3c, *t*_1/2_ = 297 ± 19 s). Interestingly, the W370F/S372H double mutant of *Xl*64 (*Xl*64-W370F/S372H), which mimics the circumstance of the Trp-triad in *At*64, exhibited ~ fourfold faster (*t*_1/2_ = 73 ± 4 s) photoreduction kinetics than *Xl*64-W370F (Fig. 3c), illustrating that the introduction of the His residue elevated the photoreduction capability via the Trp-triad in *Xl*64 lacking Trp_4_H. Together, these results suggest that the His residue next to Trp_3_H does indeed play an important role in the FAD photoreduction via the Trp-triad.

### Photoreduction experiments on other His382 mutants

**Figure 4:**
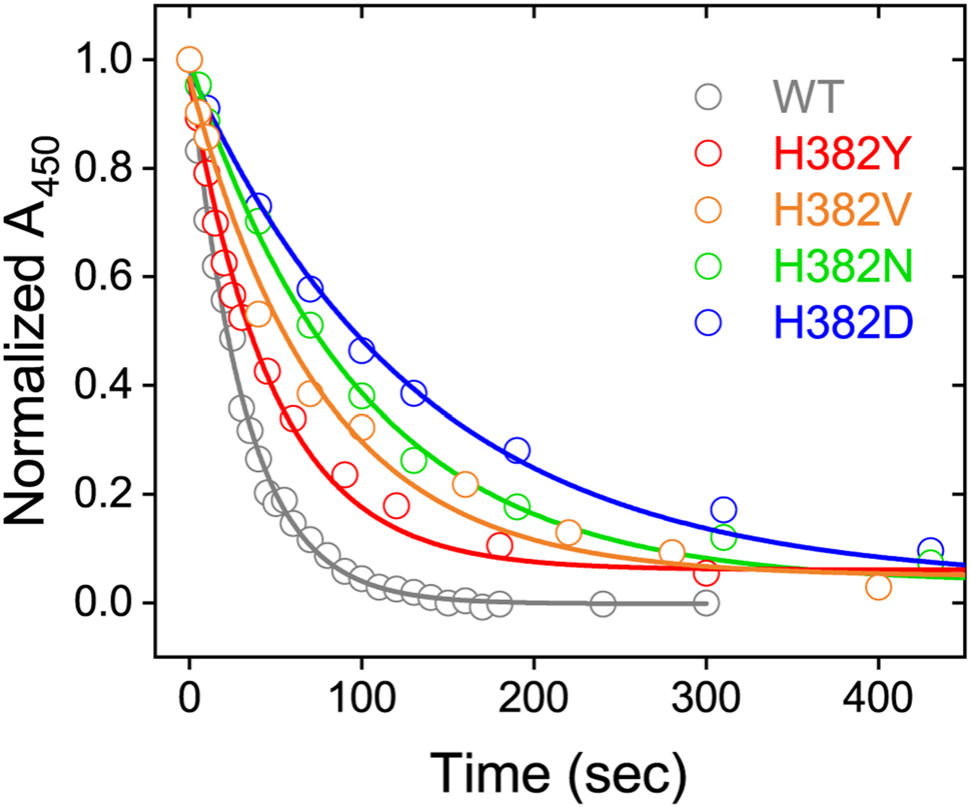
FAD photoreduction kinetics for H382Y (red), H382V (orange), H382N (green), and H382D (blue) *At*64 mutants reflected by the decay of normalized *A*_450_ values compared to WT (grey). All normalized *A*_450_ plots could be reasonably fitted by monoexponential decay functions. Spectral data for the mutants are available in Supplementary Fig. 2.

In general, His residues play a variety of roles in biological functions based on their polarity^31^, acid–base properties^32^, and the planarity/aromaticity^33^. To identify which feature of His382 in *At*64 controls the photoreduction via the Trp-triad, various His382 mutants were subjected to the photoreduction experiment. Although some mutants could not be isolated (presumably due to the loss of the structural integrity), we successfully purified the H382D, H382N, H382V and H382Y mutants of *At*64 without any apparent structural or functional perturbations as confirmed by their typical spectral changes during the photoreduction process (Supplementary Fig. 2). Their photoreduction kinetics were found to be 4.3, 3.3, 2.4, or 1.6-fold slower than *At*64-WT for the H382D, H382N, H382V, or H382Y mutant, respectively (*t*_1/2_ = 92 ± 5 s for H382D, *t*_1/2_ = 71 ± 4 s for H382N, *t*_1/2_ = 53 ± 5 s for H382V, *t*_1/2_ = 34 ± 3 s for H382Y, Fig. 4).

The slowest photoreduction kinetics of H382D among the tested mutants indicates that the capacity of His382 to act as a proton acceptor for Trp_3_H^•+^ is likely not required to improve the photoreduction efficiency. The faster kinetics of H382V *vs.* that of H382N indicates that increased polarity near Trp_3_H is not beneficial either. The most similar kinetics to *At*64-WT was observed for the H382Y mutant. This result suggests the possibility that the planarity/aromaticity or just plain bulkiness of the His and Tyr residues might be the key factor that boosts the photoreduction efficiency. Interestingly, the His → Tyr substitution is found in (6–4) PL orthologues from primitive plants such as *Lycopodiopsida* and *Bryophyta* (our Blast-p search showed that the His residue is conserved among 95% of plant (6–4) PL orthologues and the rest has a Tyr residue in this position, Fig. 2b). Altogether, the photoreduction experiments on the His382 mutants suggest that small and polar residues are not good alternatives to His382 and that the proton-accepting ability and/or polarity of His382 are not required to make the FAD photoreduction in *At*64 efficient.

### Molecular dynamics simulations suggest that the regulated solvation of Trp_3_H could play an essential role in the photoreduction of *At*64

**Figure 5:**
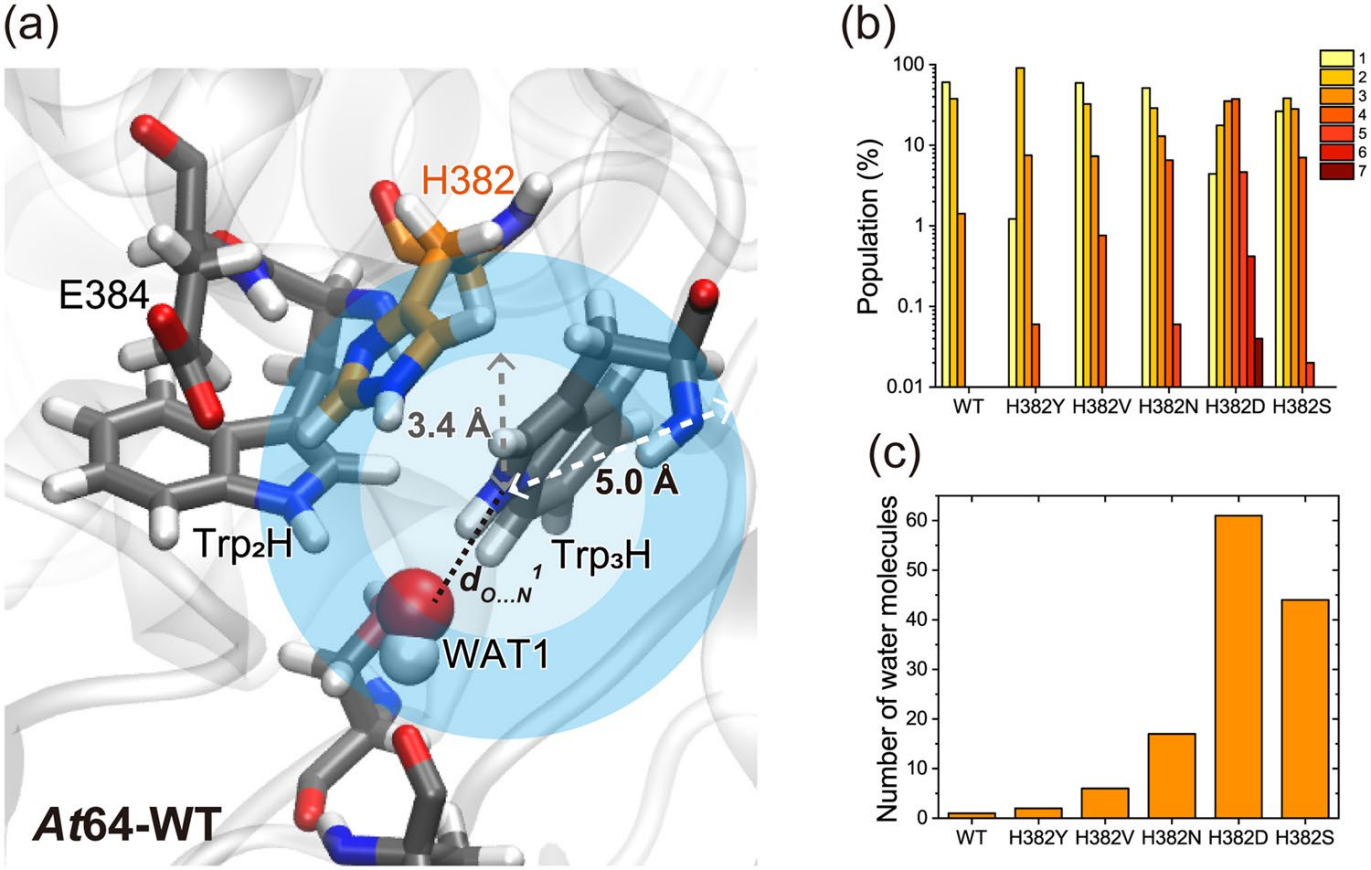
Trp_3_H solvation in WT *At*64 and its His382 mutants in MD simulations. (**a**) A snapshot of the WT structure. The first (HS1) and the second (HS2) hydration shell of the nitrogen atom of the Trp_3_H indole ring is colored in light and dark blue, respectively. For a snapshot of H382Y see Supplementary Fig. 4a. (**b**) Number of water molecules in HS2 (within 5.0 Å of the nitrogen atom of the Trp_3_H indole ring) for WT and mutant *At*64 proteins. The number of water molecules within HS2 in a frame are counted over all the frames. (**c**) Total numbers of the respective water molecules coming in and out HS1 over the 100 ns simulation time window.

Given the observations that FAD photoreduction in *At*64 significantly slowed down upon mutation of His382 to relatively compact and polar residues (Ser, Asn, and Asp), we considered the possible involvement of solvation of Trp_3_H in the photoreduction. To evaluate the solvation, we performed molecular dynamics (MD) simulations for the His382 mutants in the same way as previously reported^30^, and analyzed the presence of water molecules around the Trp_3_H in the last 100 ns of the production runs. We defined the area within 3.4 and 5.0 Å of the nitrogen atom of the Trp_3_H indole ring as the first (HS1) and the second (HS2) hydration shell, respectively (Fig. 5a). Because bulk water molecules could come into HS1 through HS2 and HS2 could be susceptible to the mutation of His382, we first counted the number of water molecules in HS2 in each frame. As expected, the water molecule distribution in HS2 demonstrated that the replacement of His382 by Ser, Asn, and Asp residues resulted in an increased solvation of Trp_3_H compared to Val, Tyr, and His residues (Fig. 5b, and Supplementary Table 1). To examine the influence of the solvation in HS2 on that in HS1, we also analyzed the water molecule distribution in HS1 (Supplementary Fig. 3), showing a clear difference between the WT protein and all the His mutants. Indeed, WT exclusively bore only one water molecule in HS1 in the course of the simulation time, while the mutants could have more than two molecules in a frame. We recently reported that a water molecule was stably captured proximal to Trp_3_H during a MD simulation for WT (WAT1 in Fig. 5a), and that the mutation of Ser412 (hydrogen-bonding to WAT1) to Ala significantly reduced the photoreducibility of *At*64^30^. As WAT1 is located in HS1, the observed single water molecule in the water molecule distribution in HS1 is assigned to be WAT1. It is conceivable that the mutation of His382 alters the coordination of WAT1, however, as shown in Supplementary Table 2, the distance between the oxygen atom of WAT1 and the nitrogen atom of the indole ring of Trp_3_H (*d*_*O…N*_^*1*^) during 100 ns simulation time for all the mutants was approximately the same as in WT. This result suggests that the mutation of His382 does not significantly affect the WAT1 coordination and that another structural aspect is likely engaged in the fine-tuning effect of His382.

Thus, we hypothesized that the presence and/or behavior of the additional water molecules (other than WAT1) in HS1 could affect the photoreduction in the His382 mutants of *At*64. To explore the dynamic behavior of water molecules, we counted the total numbers of the water molecules coming in and out of the HS1 during the 100 ns simulation, by tracing the ID of the water molecules (Fig. 5c and Supplementary Table 3). The results clearly show that there is a tendency that mutants with better water accessibility to HS1 are more difficult to photoreduce. As described above, WAT1 was the only water present in HS1 of WT, suggesting that His382 would play a role in preventing water access to Trp_3_H. Interestingly, the result for the H382Y mutant, which exhibited similar photoreduction kinetics to WT (Fig. 4), indicated that only one additional water molecule (WAT2) came into and out of HS1, and that other randomly moving water molecules outside HS1 did not enter HS1. Noteworthy, WAT2 in H382Y has been captured at the position corresponding to the place occupied by His382 in WT during the simulation time (Supplementary Fig. 4). The limited access of random water molecules into HS1 in H382Y (Fig. 5c) suggested that WAT2 prevents other water molecules from approaching Trp_3_H, in a similar way as His382 in WT. In summary, our MD simulation and photoreduction experiments indicate that a more solvent-accessible environment around Trp_3_H in the H382S, H382N, and H382D mutants could have a negative impact on their photoreduction, while the photoreduction of *At*64-WT and H382Y seems to be enhanced by regulating the solvent access to Trp_3_H.

According to the Marcus theory describing ET reactions, solvation of the reaction partners can affect the free energy difference (Δ*G*), the reorganization energy (*λ*), and the ET rate^34^. We thus estimated the effect of His382 on Δ*G* and *λ* for the ET from Trp_3_H to Trp_2_H^•+^ by classical MD simulations^35,36^ for WT and H382S (see Supplementary Methods). However, the calculated Δ*G* and *λ* values for H382S differed only slightly from those obtained for WT (Supplementary Table 4), suggesting that His382 does not significantly affect these parameters.

### Transient absorption spectroscopy on ns-µs timescales reveals that the regulated solvent accessibility impedes Trp_3_H^•+^ deprotonation in *At*64-WT

**Figure 6:**
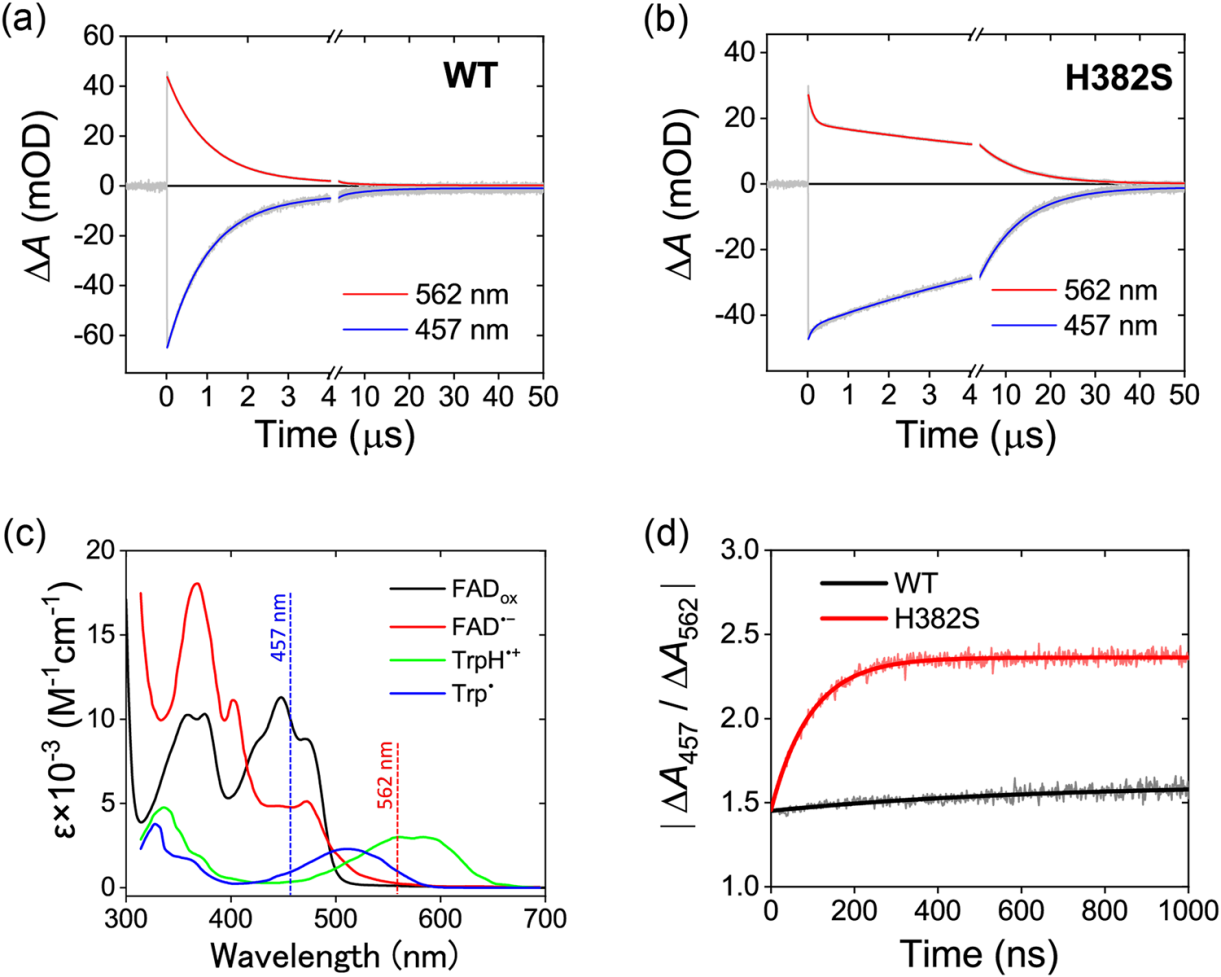
Transient absorption spectroscopy of WT and H382S *At*64 on a ns/µs timescale. Two representative wavelengths were chosen to follow the fate of the flavin and the Trp species separately: 457 nm, where the signal reflects mainly the FAD_ox_ absorption bleach due to its flash-induced reduction to FAD^•−^ and the subsequent recovery of FAD_ox_, and 562 nm, where the major contribution to absorption changes should come from the formation and decay of TrpH^•+^/Trp^•^ species. The recorded signals are shown in panels (**a**) and (**b**) in grey, results of their biexponential global fits are shown in blue (for 457 nm) and in red (for 562 nm). (**a**) Flash-induced absorption changes for ~ 125 µM *At*64-WT. (**b**) Flash-induced absorption changes for ~ 95 µM *At*64-H382S. (**c**) Reference absorption spectra of FAD and Trp species expected to contribute to light-induced absorption changes in *At*64. Spectra are taken from the literature^21^. (**d**) Absolute value of the ratio of the 457 nm to the 562 nm signals for WT and H382S *At*64. Both traces begin at ~ 1.5, suggesting that the nature of the flash-induced radical pair is the same in both proteins at time zero. The factor of ~ 1.5 is consistent with the formation of FAD^•−^ TrpH^•+^ pairs. While this ratio changes only slightly and slowly in the WT protein (in line with FAD^•−^ TrpH^•+^ recombination being the major process), the ratio evolves dramatically in the H382S mutant (with τ ~ 100 ns), mainly due to the rapid decay phase in the 562 nm signal. Increase of this ratio suggests that TrpH^•+^ is transformed into Trp^•^, which absorbs less than TrpH^•+^ at 562 nm.

In order to further clarify the effects of mutations on the fate of the photoinduced FAD^•−^ Trp_3_H^•+^ radical pair in *At*64, we compared WT with the least photoreducible mutant H382S by transient absorption spectroscopy in the ns-to-µs regime (Figs. 6a,b), where Trp_3_H^•+^ deprotonation and/or FAD^•−^ Trp_3_H^•+^/Trp_3_^•^ charge recombinations are typically observed in PCSf proteins. Based on the reference absorption spectra of the expected photoinduced species^21^ (Fig. 6c), we decided to follow the photoreaction at two selected wavelengths: at 457 nm, which is close to the maximum of the expected bleaching of the FAD_ox_ absorption band (due to its reduction to FAD^•−^) and where the Trp radicals do not contribute much to the absorption change; and at 562 nm, which is close to one of the two maxima of the TrpH^•+^ absorption band and where the absorption changes due to FAD_ox_ reduction to FAD^•−^ (and subsequent reoxidation upon radical pair recombination) are expected to be relatively small.

Upon excitation by ~ 5 ns flashes at 480 nm, bleaching at 457 nm and absorption increase at 562 nm with the same initial ratio of the two signals of ~ –1.5:1 (consistent with the formation of FAD^•−^ TrpH^•+^ radical pairs) in both protein samples were induced, followed by a decay of all signals to zero within less than 50 µs (Figs. 6a,b), suggesting that all photoinduced radicals recombined in this time window. However, the signal decays were markedly different in the two proteins: while the decay of the WT signals at both wavelengths was essentially monoexponential with a time constant τ_1_ of ~ 1 µs (according to the fit, traces of a slower τ_2_ ~ 8 µs process amounting to less than 10% of the signal amplitudes were present), the decay of the H382S signals was clearly biexponential, with τ_1_ of ~ 100 ns and τ_2_ of ~ 9.5 µs. To qualitatively describe the processes occurring with these kinetics, we analyzed the amplitude ratio of the signals at 457 and 562 nm (|Δ*A*_457_/Δ*A*_562_|; Fig. 6d). This ratio remains constant in the case of a simple radical pair recombination, but it is sensitive to changes in the chemical nature of the radicals. TrpH^•+^ cation radical absorbs significantly more than the neutral Trp^•^ radical at 562 nm (see Fig. 6c), and TrpH^•+^ deprotonation hence leads to absorption decrease at this wavelength and to a corresponding increase in the |Δ*A*_457_/Δ*A*_562_| value. In the case of *At*64-WT, the amplitude ratio (Fig. 6d) changes only very little, indicating that the major process behind the ~ 1 µs decay of signals in Fig. 6a is charge recombination of the initially produced FAD^•−^ TrpH^•+^ pairs. The deprotonation of TrpH^•+^ radicals is apparently very slow in *At*64-WT and can hence compete with FAD^•−^ TrpH^•+^ recombination only to a very limited extent. On the other hand, the amplitude ratio in the H382S mutant dramatically increases within the first 300 ns (with the time constant of ~ 100 ns), indicating that TrpH^•+^ deprotonation occurring within this kinetic phase is much faster in H382S (Fig. 6d). After the initial growth phase, the |Δ*A*_457_/Δ*A*_562_| value stabilizes at ~ 2.4. The following decay of the transient absorption signals in ~ 9.5 µs (Fig. 6b) hence reflects pure recombination of the FAD^•−^ Trp^•^ pairs.

Analysis of the phase amplitudes obtained from the biexponential fits of the signals from *At*64-WT confirms that > 90% of the FAD^•−^ TrpH^•+^ radical pairs directly recombine within the ~ 1 µs kinetic phase (the amplitudes of this phase are in a good agreement with the difference spectrum of FAD^•−^ – FAD_ox_ + TrpH^•+^, see Supplementary Fig. 5a). The remaining < 10% of pairs, in which tryptophan cation radicals do deprotonate, decay in ~ 8 µs. Indeed, the amplitudes of this minor phase fit well the difference spectrum of FAD^•−^ – FAD_ox_ + Trp^•^ (Supplementary Fig. 5a). In *At*64-H382S, the situation is markedly different. Since the TrpH^•+^ cation radicals rapidly deprotonate within the first ~ 100 ns kinetic phase (the amplitudes of this phase are consistent with the TrpH^•+^ – Trp^•^ difference spectrum, see Supplementary Fig. 5b), only a very small fraction of FAD^•−^ TrpH^•+^ pairs recombine directly (in competition with the fast deprotonation). The fraction of the remaining FAD^•−^ Trp^•^ pairs (Supplementary Fig. 5b) is hence much larger in H382S than in the WT protein. Nevertheless, the lifetimes of the FAD^•−^ Trp^•^ pairs are comparable in both proteins (~ 8 µs in WT *vs.* ~ 9.5 µs in H382S).

Given that the ~ 1 µs FAD^•−^ TrpH^•+^ decay phase in WT reflects to ~ 90% recombination and to ~ 10% TrpH^•+^ deprotonation, the intrinsic time constant for TrpH^•+^ deprotonation is ~ 10 µs in this protein. This means that while the H382S mutation has virtually no impact on the lifetime of the FAD^•−^ Trp^•^ pairs (see above), it accelerates the TrpH^•+^ deprotonation by a factor of ~ 100 (from ~ 10 µs in WT to ~ 100 ns in H382S) and shortens the lifetime of the FAD^•−^ TrpH^•+^ pairs by a factor of ~ 10 (from ~ 1 µs in WT to ~ 100 ns in H382S). To our knowledge, Trp_3_H^•+^ deprotonation in *At*64 in ~ 10 µs is the slowest terminal TrpH^•+^ deprotonation rate ever reported for a WT PCSf protein (the second-slowest being the Trp_4_H^•+^ deprotonation in *Xl*64 in ~ 2.5 µs under very similar conditions^21^).

When estimating the energy of the excitation pulses using the [Ru(bpy)_3_]^2+^ actinometer^37^, we also estimated the quantum yield (see SI) of the photoinduced FAD^•−^ TrpH^•+^ pairs detected at ‘time zero’ of our experiment, *i.e.*, pairs that had not been lost due to ultrafast recombination processes faster than our time resolution (*i.e.* ~ 5 ns, limited by the excitation pulse length). The yield of the detected FAD^•−^ TrpH^•+^ radicals is ~ 80% in both *At*64-WT and -H382S, which means that the losses due to ultrafast FAD^•−^ TrpH^•+^ recombination are only ~ 20%. The similar quantum yield of stable FAD^•−^ Trp_3_H^•+^ pairs in *At*64-WT and -H382S indicates that His382 does not have a significant impact on the electron transfer through the Trp triad in *At*64.

## Discussion

Upon photoactivation, *A. thaliana* (6–4) photolyase exhibits by far the slowest (~ 10 µs) deprotonation of the terminal TrpH^•+^ cation radical of all PCSf proteins studied to date. In the absence of extrinsic reductants in vitro, the consequence of this slow deprotonation rate is that most (~ 90%) of the photoinduced FAD^•−^ Trp_3_H^•+^ radical pairs recombine as such (in ~ 1 µs) and the remaining ~ 10% of FAD^•−^ Trp_3_^•^ pairs (in which Trp_3_H^•+^ has been deprotonated) recombine in ~ 8 µs. Putting the ~ 10 × faster FAD photoreduction in *At*64-WT (than in H382S) in vitro (Fig. 3a) and the ~ tenfold higher survival rate of WT-expressing *E. coli* cells in the repair activity assay (compared to cells expressing *At*64-H382S; Fig. 3b) into context with these results, it seems that the extrinsic reductants act much more easily upon TrpH^•+^ than upon Trp^•^, both in vitro and in vivo. This is likely because TrpH^•+^ reduction requires a mere ET, while Trp^•^ reduction requires transfer of not just an electron but also of a proton (or of a hydrogen atom H^•^) and because ET can occur over longer distances than the transfer of a proton or of a hydrogen atom. In any case, formation of a much greater fraction (compared to WT) of the longer-lived FAD^•−^ Trp^•^ pairs in H382S does not seem to be able to compensate to any visible extent for the ~ 10 × shortened lifetime of the FAD^•−^ TrpH^•+^ pairs in this mutant. The slow deprotonation of the terminal tryptophan cation radical and the consequently extended lifetime of the FAD^•−^ Trp_3_H^•+^ pair (and likely also that of the FADH^−^ Trp_3_H^•+^ pair in the second photoactivation step) considerably enhance the yield of *At*64 photoactivation (and hence the rate of FAD photoreduction under continuous illumination)—by providing the extrinsic reductants more time and opportunity to encounter and quench Trp_3_H^•+^ at the protein surface and thereby stabilize the reduced flavin in the protein interior by preventing recombination with its radical counterpart.

Searching for the cause of the unusually slow Trp_3_H^•+^ deprotonation, we identified a neighboring histidine residue, which is highly conserved in plant (6–4) PLs (His382 in *At*64 numbering), and the mutation of which (to diverse alternative residues—Tyr, Val, Asn, Asp and/or Ser) indeed had a significant negative effect on the rate of *At*64 photoactivation in vitro. In the case of the in vivo-tested (and the least in vitro-photoactivatable) H382S mutant, the mutation also had a dramatically deleterious impact on the survival of UV-irradiated and Vis-reactivated *E. coli* cells, suggesting that the His382 residue next to the terminal (3rd) tryptophan likely plays an important role also in the in-vivo photoactivation of *At*64 via the Trp chain.

**Figure 7:**
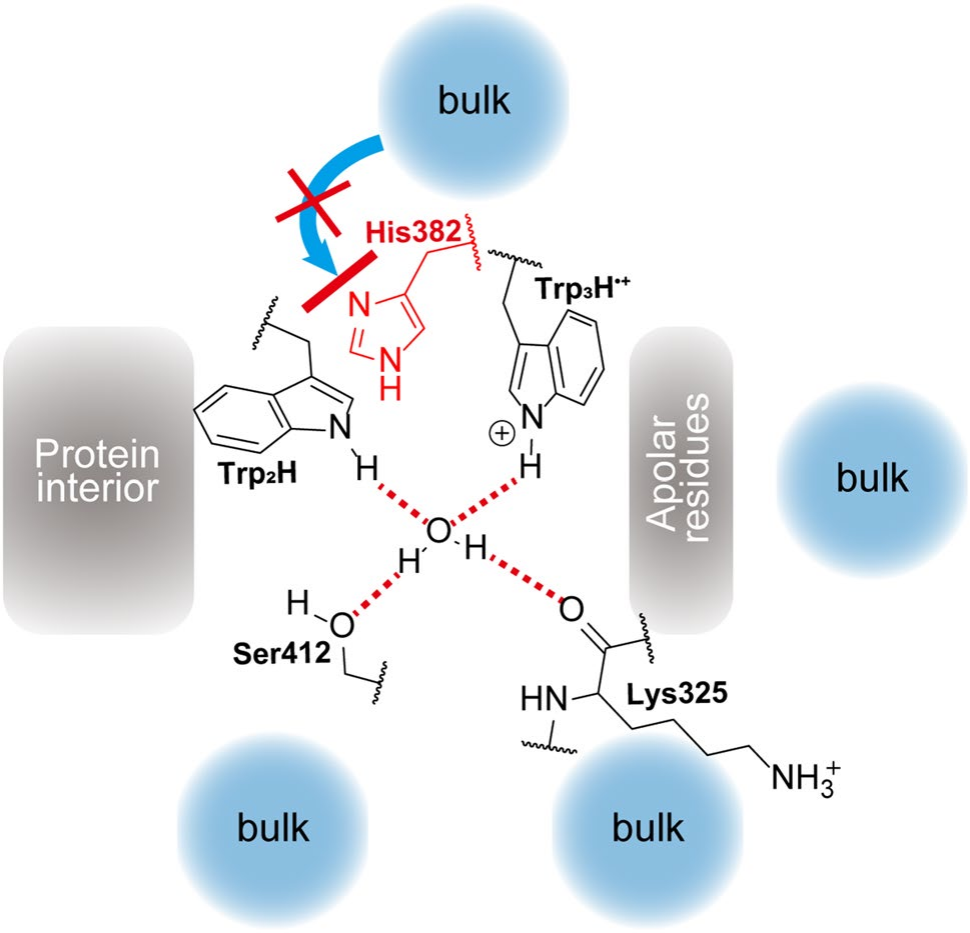
Schematic model showing how the surroundings of Trp_3_H^•+^ prevent the proton release to the solvent. The nitrogen atom of Trp_3_H^•+^ is encircled by the coordinated water molecule WAT1, non-polar residues (including I327 and F380 shown in Fig. 2), and His382 (highlighted in red). WAT1 does not interact with and cannot escape to the bulk because it is fully restrained by the hydrogen bond interactions (indicated by dashed red lines) with the residues of the surrounding protein.

According to our MD simulations, the native His382 blocks access of solvent molecules to the nitrogen atom of Trp_3_H^•+^ in *At*64 (Fig. 7), and it does so much more efficiently than any alternative amino acid in the tested mutants. In the WT protein, the only water molecule interacting with Trp_3_H^•+^, referred to as WAT1 in this text, is tightly coordinated by other amino acids within *At*64. It never leaves the first hydration shell of the nitrogen atom of Trp_3_H^•+^ and it does not interact with the bulk buffer enough to provide a functional channel for a proton transfer from Trp_3_H^•+^ to the buffer.

Finally, the efficiency of *At*64 photoactivation is further enhanced by exceptionally low losses (~ 20%) due to ultrafast (< 5 ns) recombination of the photoinduced FAD^•−^ Trp_1_H^•+^ and/or FAD^•−^ Trp_2_H^•+^ radical pairs. This is significantly less than in any other PCSf protein studied so far. For comparison, in *E. coli* CPD PL ~ 35% of the photoinduced radical pairs are lost within the first few ns^38^, in *Chlamydomonas reinhardtii* animal-like CRY it is ~ 50%^25^, in *Xl*64 ~ 75%^21^, and in *At*CRY 80 to 95%, depending on the presence or absence of ATP^17^. In *Dinoroseobacter shibae* NewPHL, which is an ancestral PL containing a mere Trp dyad, as much as ~ 90% of the photoinduced radical pairs are lost within the first few nanoseconds and most of the remaining ~ 10% pairs recombine within the next ~ 50 ns^39^. Interestingly, our structural comparison between *At*64 and *Xl*64 does not show any remarkable difference in the residues within 4 Å of FAD and Trp_1_H (Supplementary Fig. 6a), but the residues around Trp_2_H are different. Notably, an arginine residue (Arg387) is located near Trp_2_H in *At*64, which is replaced by glutamine (Gln377) in *Xl*64 (Supplementary Fig. 6a). This arginine is highly conserved in plant (6–4) PL orthologues but is not found in any animal (6–4) PL (Supplementary Figs. 6b,c). The positively-charged guanidinium moiety of Arg387 could destabilize the localization of the hole on Trp_2_H and accelerate the successive electron transfer from Trp_3_H, leading to the ultrafast charge separation between FAD and Trp_3_H and low recombination losses. This hypothesis will be addressed by future experimental and computational studies.

## Methods

### Plasmid construction

For the (6–4)PP repair activity assay in bacterial cells^5,29,40^ and protein production, pGEX-4T-1 and pET28a( +) plasmids that produce the (6–4) PLs were constructed, respectively. First, the desired mutations (W329F and H382S) of *At*64 were introduced into the pGEX-4T-1 plasmid carrying the *At*64-WT gene^29^ with the QuikChange Site-Directed Mutagenesis Kit. The PCR primers used for the mutagenesis are shown in Supplementary Table 5 (Entry 1 and 4). After sequencing, the obtained plasmids were used for the assay.

The mutant genes in the pGEX plasmids were then amplified and subcloned into the *Nde*I/*Xho*I restriction site of the pET-28a( +) vector with the specific primers as shown in Supplementary Table 5 (Entry 8). The H382D, H382N, and H382V mutations of *At*64 were also introduced into the pET28a vector carrying the *At*64-H382S gene by the QuikChange Site-Directed Mutagenesis kit (Supplementary Table 5, Entry 2, 3, and 5). As for the H382Y mutant, the 5’ upstream and 3’ downstream of the mutation site were separately amplified using the primers shown in Supplementary Table 5 (Entry 6), and the amplicons were fused into the pET-28a( +) vector linearized with the *Nde*I and *Xho*I treatment, by using the In-fusion HD Cloning Kit (Takara). The S372H mutation of *Xl*64 was introduced into the pET28a( +) vector carrying the reported *Xl*64-W370F gene^29^ using the In-fusion HD Cloning kit with the specific primers shown in Supplementary Table 5 (Entry 7). The obtained plasmids were sequenced and used for the protein production.

### (6–4)PP repair activity assay in bacterial cells

The *E. coli* SY32 strain (*uvrA*^−^, *recA*^−^, *phr*^−^), in which CPD PL activity is rescued by the pACYC184 plasmid coding *E. coli* CPD PL gene, has been used for (6–4)PP repair activity assay in bacterial cells^30^. SY32 cells were transformed with a pGEX-4T-1 plasmid expressing a (6–4) PL variant and the colonies were selected on Luria broth (LB) agar plates containing tetracycline (10 µg mL^−1^) and ampicillin (80 µg mL^−1^). The transformant was cultivated in a 1.5 mL of LB medium containing tetracycline and ampicillin at 37 °C overnight. The culture was diluted to OD600 = 0.5 with an LB medium. The diluted culture was induced with 2.4 µL of 10 mg mL^−1^ isopropyl-β-D-thiogalactoside (IPTG) and shaken at 37 °C for 1 h. The culture was appropriately diluted with phosphate-buffered saline, and 150 µL of aliquots were spread onto LB agar plates containing tetracycline and ampicillin. The plates were irradiated with 20 W UV germicidal lamp (UVL20PH-6, Sen Lights Co Ltd., Osaka, Japan) through metal mesh filters (2.0 µW cm^−2^, calibrated with a UVX radiometer equipped with a 254 nm probe, UVP, LLC, Upland, CA) for 15 or 30 s to yield a total irradiance of 0.3 or 0.6 J m^−2^, respectively. The plates were subsequently illuminated with fluorescent lamps (18 W × 4, FL20SSD/18, Toshiba, Tokyo, Japan) for 30 min, and then incubated at 37 °C overnight. After incubation, the number of colonies was counted taking into account the dilution percentage. All survival rates were normalized to the number of colonies obtained without UV irradiation. The experiments were independently performed in triplicate (*n* = 3), and the statistical significance was analyzed with a Student’s *t*-test. The significant cutoff value was set to 0.05.

### Protein purification

For purification of *At*64 variants, we modified a previously reported expression and purification protocol^41^. *E. coli* C41 (DE3)/pLysS (Lucigen) cells were transformed with a pET-28a( +) plasmid encoding the (6–4) PLs gene and grown in a 2 L of LB medium containing kanamycin (20 µg mL^−1^) in a 5 L flask with baffles at 37 °C. When OD600 reached 1.2, the culture was cooled to 25 °C. Protein production was then induced with a final concentration of 0.2 mM IPTG, and the culture was further incubated at 25 °C for 24 h. After harvest, the pellet was frozen by liquid nitrogen and thawed on ice. The cells were resuspended in 40 mL of a lysis buffer (20 mM NaH_2_PO_4_, 500 mM NaCl, 5% glycerol, pH 7.4 adjusted by KOH, plus 65 mg of lysozyme) and lysed by sonication. The cell lysate was centrifuged, and the supernatant was loaded onto an open column filled with TALON Metal Affinity Resin (Clontech, TaKaRa) equilibrated with the lysis buffer. Proteins non-specifically bound to the resin were washed out with four column volumes of a wash buffer (20 mM NaH_2_PO_4_, 500 mM NaCl, 10 mM imidazole, 5% glycerol, pH 7.4 adjusted by KOH), and the His-tagged protein was eluted with an elution buffer (20 mM NaH_2_PO_4_, 500 mM NaCl, 500 mM imidazole, 5% glycerol, pH 7.4 adjusted by KOH). For further purification, the green eluate was loaded onto a HiTrap Heparin HP column (GE Healthcare) and purified with a step-gradient of 100–500 mM NaCl in a buffer containing 50 mM Tris–HCl and 5% glycerol (pH 8.0). The purified protein was confirmed by 10% SDS-PAGE, and its concentration was measured based on the FAD absorbance at 450 nm using a molar extinction coefficient of 11 300 L mol^−1^ cm^−1^.

### Measurement of steady-state photoreduction kinetics by UV–Vis absorption spectroscopy

Steady-state photoreduction of recombinantly produced proteins was performed under anaerobic conditions as previously described^30^. 70 µL of protein diluted to 20 µM was applied to a Micro Bio-Spin 6 column (BIO-RAD) equilibrated with a reaction buffer (20 mM phosphate, 500 mM NaCl, 10% glycerol, pH 7.5). An aliquot (60 µL) of the eluate was transferred into an anaerobic 10 × 2 × 8 mm (length × width × height) inner volume quartz cuvette (Starna, 16.160-F/4/Q/10 GL 14/2/Z15). After the cuvette was sealed with a screw cap and PTFE-coated silicone and rubber septa, the air inside the cuvette was replaced with nitrogen through the septa. Further preparation was performed in an anaerobic glovebox. The sample was mixed with L-cysteine (the final concentration was 5 mM) and the reaction buffer up to a total volume of 240 µL under anaerobic and dark conditions.

The anaerobic samples were illuminated with continuous light (430–800 nm) from a MAX-150 xenon lamp (Asahi Spectra) through the 10 mm × 8 mm window on ice (illumination time varied depending on the sample). After shaking the sample gently, an absorption spectrum was recorded through the 10 mm path by a UV–Vis spectrometer Lambda 35 UV–Vis spectrometer (PerkinElmer) or V-730 spectrometer (JASCO)]. The absorbance at 450 nm of the obtained spectra was plotted and fitted with a monophasic exponential decay function with the Origin2019 software.

### Transient absorption spectroscopy

The proteins were dissolved in a buffer consisting of 50 mM Tris–HCl (pH 8.0), 500 mM NaCl, and 5% glycerol, at final concentrations of ~ 125 µM for *At*64-WT and ~ 95 µM for *At*64-H382S. The transient absorption setup has been described in detail elsewhere^21,26,38^. Nd:YAG-pumped optical parametric oscillator (OPO; Brilliant B/Rainbow; ∼5 ns, 480 nm, ∼2 mJ cm^−2^) was used as an excitation source. The laser energy was estimated from transient absorption signals using [Ru(bpy)_3_]^2+^ as an actinometer^37^. Monitoring light at two selected wavelengths (457 and 562 nm) was provided by continuous-wave lasers (Cobolt Twist™ and Oxxius 561–25-COL-002, respectively). The measuring light was perpendicular to the excitation laser beam and passed through the sample along the 10-mm path of a 2 × 2 × 10 mm (W × H × L) quartz cell with self-masking solid black walls (Starna). Flash-induced changes of the transmission of the sample were monitored behind the sample by a Si photodiode (Alphalas UPD-500-UP, < 500 ps rise time) coupled to a Tektronix MSO 64 digital oscilloscope with bandwidth limit set to 200 MHz. All shown traces are averages of 16 signals recorded with a repetition rate of 2 Hz. The samples were measured at room temperature. Transient absorption kinetics were determined using the Levenberg–Marquardt least-squares optimization algorithm in Origin 2020 (by OriginLab), globally fitting the trend lines according to the equation: *y*(*t*) = *A*_1_ × e^−*t/*τ1^ + *A*_2_ × e^−*t*/τ2^ + *y*_0_.

## Supplementary Information

Supplementary Information.
