# Supplementary material for: Limited solvation of an electron donating tryptophan stabilizes a photoinduced charge-separated state in plant (6–4) photolyase
Source: Sci Rep. 2022 Mar 24;12:5084. doi: 10.1038/s41598-022-08928-0 (PMC8948257; doi:10.1038/s41598-022-08928-0)
Supplement: Supplementary file 1 — Supplementary Information. [file 41598_2022_8928_MOESM1_ESM.pdf]

*Supplementary Information*

# Limited solvation of an electron donating tryptophan stabilizes a photoinduced charge-separated state in plant (6-4) photolyase

Yuhei Hosokawa,[1] Pavel Müller,[2] Hirotaka Kitoh-Nishioka,[3] Shigenori Iwai,[1] and Junpei Yamamoto[1,*]

[1]Graduate School of Engineering Science, Osaka University, 1-3 Machikaneyama, Toyonaka, Osaka 560-8531, Japan.

[2]Université Paris-Saclay, CEA, CNRS, Institute for Integrative Biology of the Cell (I2BC), 91198, Gif-sur-Yvette, France.

[3]Graduate School of System Informatics, Kobe University, 1-1 Rokkodai, Nada-ku, Kobe 657-8501, Japan.

*To whom correspondence should be addressed.

Junpei Yamamoto – Tel. and Fax: +81 6 6850 6240; E-mail: yamamoto@chem.es.osaka-u.ac.jp

**Supplementary Methods**

*Multiple sequence alignment*

For assignment of plant and animal (6-4) PL orthologues, we performed blastp[1] searches using the *At*64 and *Xl*64 amino acid sequences as representative plant and animal (6-4) photolyase sequences against the refseq_protein database with an *E*-value threshold of $10^{-150}$. The resulting sequences (202 sequences for plants and 1471 sequences for animals) were aligned with COBALT[2]. From the multiple sequence alignments, the WebLogo[3] was created with the version 3.7.4.

*Estimation of the excitation energy and of the quantum yield of 'stable' $FAD^{\bullet-}$ $Trp_3H^{\bullet+}$ pairs detected by transient absorption spectroscopy*

The energy of laser flashes entering the *At*64 samples in the transient absorption spectroscopic experiments (Fig. 6) was estimated using the $[Ru(bpy)_3]^{2+}$ actinometer[4-5] under the same excitation conditions and geometry. The sample containing 32.3 µM $[Ru(bpy)_3]Cl_2$ had an absorbance $A_{480}$ of 0.2164 over the 1 cm path and 0.0433 over the 2 mm path at the excitation wavelength (480 nm). $A_{480}$ of 0.0433 converts to a transmittance $T_{480}$ of 90.51%, *i.e.* 9.49% of the excitation light was absorbed by the actinometer over the 2 mm path. The flashes induced an absorption change of $\Delta A_{457}$ = 0.0465 (recorded over 1 cm optical path; 64 signals were averaged). Assuming that the $\Delta\varepsilon_{457}$ value for the formation of the $^3$MLCT (metal-to-ligand charge transfer state formed with a ~100% quantum yield) is close to the $-1.1 \times 10^4$ $M^{-1}cm^{-1}$ as estimated for 450 nm[5] (which is reasonable given the shape of the difference spectrum[6]), one obtains a concentration of the excited $[Ru(bpy)_3]^{2+}$ complexes / formed $^3$MLCT states of ~4.23 µM. In the excited volume of 40 µL, this concentration corresponds to ~$1.69 \times 10^{-10}$ mol of absorbed photons (out of the total ~$1.78 \times 10^{-9}$ mol, given that only 9.49% photons were absorbed). Since 480 nm photons have an energy of 249 220 J/mol, the energy absorbed by the $[Ru(bpy)_3]Cl_2$ actinometer was ~42 µJ. Considering that the window through which the sample was excited had a surface of 0.2 $cm^2$ (0.2 × 1.0 cm) and that only 9.49% of the excitation light was absorbed, one obtains an excitation energy (per pulse and per $cm^2$) of 2.23 mJ.

The ~125 µM *At*64-WT sample had an absorbance $A_{480}$ of 1.0334 over the 1 cm path and 0.2067 over the 2 mm path at the excitation wavelength. $A_{480}$ of 0.2067 converts to a transmittance $T_{480}$ of 62.13%, so 37.87% of the ~$1.78 \times 10^{-9}$ mol photons entering the sample (*i.e.* ~$6.74 \times 10^{-10}$ mol) were absorbed by the *At*64-WT sample over the 2 mm path. The maximum concentration of the flash-induced radical pairs in the excited volume of 40 µL in the hypothetical case of a 100% quantum yield would hence be ~16.9 µM. $\Delta\varepsilon_{457}$ corresponding to the formation of a $FAD^{\bullet-}$ $TrpH^{\bullet+}$ radical pair equals $\varepsilon_{457}$ ($FAD^{\bullet-}$) – $\varepsilon_{457}$ ($FAD_{ox}$) + $\varepsilon_{457}$ ($TrpH^{\bullet+}$) ≅ (4740 – 9700 + 350) $M^{-1}$ $cm^{-1}$ = –4610 $M^{-1}cm^{-1}$ (TrpH does not absorb at 457 nm). The observed initial amplitude of the *At*64-WT signal at 457 nm

$\Delta A_{457\ (t\to0)}$ = –0.065 hence corresponds to ~14.1 µM $FAD^{\bullet-}$ $TrpH^{\bullet+}$ pairs, which is ~83% of the maximum of pairs that could have hypothetically been formed by the excitation flash-.

Even though we do not know some of the $\varepsilon$ values precisely because the absorption spectra of the $FAD^{\bullet-}$ and $TrpH^{\bullet+}$ radicals can (and do) slightly vary from protein to protein, the quantum yields of most of the other PCSf proteins mentioned in the main text were determined by the same method (and essentially confirmed by later ultrafast experiments), so it is safe to say that the losses due to ultrafast recombination of the $FAD^{\bullet-}$ $Trp_1H^{\bullet+}$ and $FAD^{\bullet-}$ $Trp_2H^{\bullet+}$ radical pairs are indeed significantly lower in *At*64 than in the other studied PCSf proteins and amount to ~20%.

When the vertical axes are scaled to reflect the difference in protein (FAD) concentrations (Fig. 6 of the main text), the initial amplitudes of the signals obtained for the H382S mutant are practically the same as for the WT protein, indicating that the quantum yield of the 'stable' $FAD^{\bullet-}$ $TrpH^{\bullet+}$ pairs (and the losses through their ultrafast recombination) are very similar in both proteins. Using the same method of calculation as for the WT protein, we estimate that the excitation of the ~95 µM *At*64-H382S, with $A_{480}$ = 0.1545 over the 2 mm path and $\Delta A_{457\ (t\to0)}$ = –0.048 over the 1 cm path, yielded ~10.4 µM $FAD^{\bullet-}$ $TrpH^{\bullet+}$ pairs, which is ~78% of the maximum ~13.3 µM of pairs that would have been formed in a hypothetical case of a 100% quantum yield.

*Molecular dynamics simulations for solvation analyses*

Molecular dynamics (MD) simulations for solvation analyses were conducted with the AMBER 16 program package[7], as reported previously for the simulation of *At*64-WT[8]. The initial structures of H382D, H382N, H382S, H382V, and H382Y mutants of *At*64 were generated with SWISS-MODEL[9] using the *At*64-WT crystal structure (PDB entry: 3FY4) as the template structure. We applied the Amber force field 14SB[10] and the previously prepared Amber force field[8] for the proteins and $FADH^-$, respectively. We solvated the proteins with TIP3P water model[11] in the simulation boxes with a margin of 12 Å from the proteins to the box boundaries and neutralized the system by adding some counter ions ($Cl^-$). In the following MD simulations, the SHAKE algorithm was used for the constraints[12], the periodic boundary condition with the particle mesh Ewald method was applied[13], and the simulation time step was set to 2 fs. The energy minimization for each system with the Sander module was performed for 5,000 steps with 10 kcal $mol^{-1}$ $Å^{-2}$ of restrictions on heavy atoms, and for 10,000 steps without any restrictions. After the system temperature was increased from 0 to 300 K for 100 ps with the NVT ensemble ($T$ = 300 K), the system was equilibrated for 1 ns with the NPT ensemble ($P$ = 1 atm and $T$ = 300 K). Then, 200 ns MD simulation in the NPT ensemble ($P$ = 1 atm and $T$ = 300 K) was performed. The last 100 ns of the trajectory for each system was recorded every 20 ps and subjected to our analyses. For *At*64-WT, the last 100 ns of the trajectory previously simulated in the same way[8] was used for our analyses.

The time-development of an atomic distance was calculated with the distance command in the CPPTRAJ module of the AMBER 16 program package[14]. For solvation analyses, we defined the area within 3.4 and 5.0 Å of the nitrogen atom of the $Trp_3H$ indole ring as the first hydration shell (HS1) and second hydration shell (HS2). The number of water molecules in HS1 and HS2 at every recording time-points was counted with the watershell command in CPPTRAJ. The behavior of the water molecules in the hydration shells was traced by identifying their IDs with the closest command in CPPTRAJ.

*Molecular dynamics simulations for estimation of electron transfer parameters*

We estimated electron transfer parameters in the photoreduction of the fully oxidized FAD ($FAD_{ox}$) in *At*64-WT and H382S mutant through the Trp-triad chain by using classical MD simulations. The force field parameters for FAD were prepared by using the Antechamber module in AMBER18[15] with GAFF2 (general AMBER force field version 2.0)[16]. The partial atomic charges of the oxidized and reduced forms of FAD ($FAD_{ox}$ and $FAD^{\bullet-}$) were evaluated by the RESP (restricted electrostatic potential) fitting scheme[17] to the B3LYP/cc-pVDZ results. The electronic structure calculations were performed using the Gaussian 16 Revision B.01 suite of programs[18]. In the fitting, we imposed the constraint condition on the RESP charges of $FAD^{\bullet-}$ identical to those of $FAD_{ox}$ except for the isoalloxazine-ring part. The atomistic coordinates of *At*64-WT and its H382S mutant were prepared using *At*64-WT crystal structure (PDB entry: 3FY4) like in the previous section. We set the force-field parameters of protein and the crystal water molecules to ff14SB[10] and TIP3P[11], respectively. In addition, the partial atomic charges of the oxidized Trp for $Trp_2H^{\bullet+}$ and $Trp_3H^{\bullet+}$ were evaluated by the RESP fitting scheme to the B3LYP/cc-pVDZ results, where we imposed the constraint condition on the RESP charges identical to those of the neutral Trp except for the side chain. We solved the constructed models of the *At*64-WT and H382S mutant in a truncated octahedron box of TIP3P water molecules having an edge distance of 12.0 Å from the protein. We added six counter ions ($Cl^-$) for electroneutrality.

First, we set the charge states of FAD and the Trp-triad to $FAD_{ox}$ and neutral Trp, respectively, for both the simulation systems of the *At*64-WT and H382S mutant. We performed the energy minimization (15,000 steps) for each system with the PMEMD module in the Amber18 suite; we imposed 50 kcal $mol^{-1}$ $Å^{-2}$ of harmonic restrictions on all the heavy atoms during the first 5,000 steps. Then, we did the following MD simulations by using GPU-accelerated PMEMD module[19–21] in the Amber18 suite with the SHAKE algorithm, the particle mesh Ewald method, a non-bonding cut-off distance of 10 Å, and a time step of 1 fs. We slowly heated both systems up to 300 K within 300 ps and kept them within the following 200 ps in the NVT ensemble, while maintaining 50 kcal $mol^{-1}$ $Å^{-2}$ of restrictions on the heavy atoms of protein and FAD. During the first equilibration phase, we did

five-successive 200 ps MD simulations in the NPT ensemble by gradually decreasing the harmonic restrictions (50, 20, 10, 5, and 1 kcal $mol^{-1}$ $Å^{-2}$). During the second equilibration phase, we did the additional 100 ns MD simulations in the NPT ensemble without any restrictions. At the beginning of the third equilibration phase, we changed the charge states of FAD and the Trp-triad; one set was $FAD^{•-}$ and $Trp_2H^{•+}$ ($Trp_1H$ and $Trp_3H$ are neutral) and the other one $FAD^{•-}$ and $Trp_3H^{•+}$ ($Trp_1H$ and $Trp_2H$ are neutral). The former and latter charge states correspond to the initial and final states of the electron-transfer from $Trp_3H$ to $Trp_2H^{•+}$ (or the hole transfer from $Trp_2H^{•+}$ to $Trp_3H$). During the third equilibration phase, we did 100 ns MD simulations on the initial and final ET states of the *At*64-WT and its H382S mutant systems in the NPT ensemble. Finally, during the subsequent 100 ns production MD simulations in the NPT ensemble, we collected the snapshots every 10 ps for the following free energy difference ($\Delta G$) and reorganization energy ($\lambda$) calculations.

Within the linear response approximation, one can evaluate the $\Delta G$ and $\lambda$ in the Marcus rate formula from the ensemble of the vertical energy differences between the initial ($i$) and final ($f$) ET states, $\Delta E_{if}$, as follows:[20,21]

$$\Delta G = \frac{1}{2}\left(\langle \Delta E_{if} \rangle_i + \langle \Delta E_{if} \rangle_f\right),$$

$$\lambda = \frac{1}{2}\left(\langle \Delta E_{if} \rangle_i - \langle \Delta E_{if} \rangle_f\right).$$

Here, $\langle\ \rangle_i$ and $\langle\ \rangle_f$ represent the thermal averages over the initial and final ET states, respectively. Since the value of $\lambda$ in the above equation includes only an outer-sphere contribution, this study ignored the inner-sphere contribution to $\lambda$. The resultant $\Delta G$ and $\lambda$ values for the *At*64-WT and H382S mutant are listed in Supplementary Table 4. It should be noted that our calculations overestimate the values of $\lambda$ because of the use of the non-polarizable force fields (ff14SB and TIP3P) and the lack of the non-ergodic effects (see the details in Supplementary Reference 22).

**Supplementary Figures and Tables**

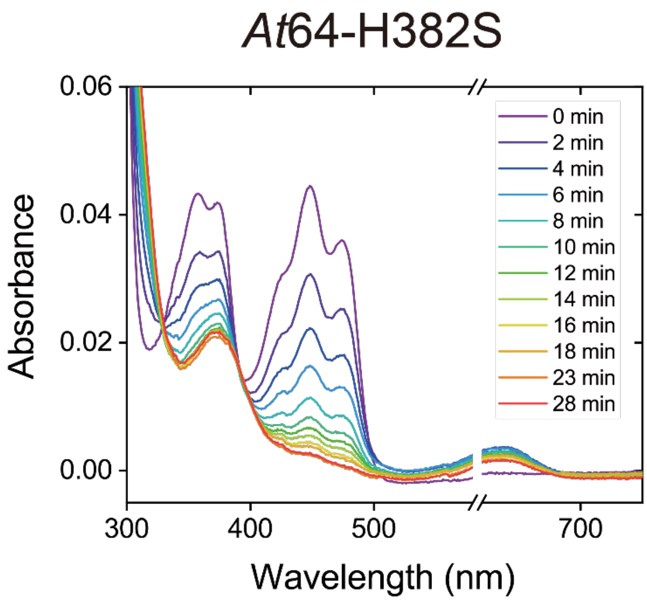


**Supplementary Fig. 1** Evolution of absorption spectra upon FAD photoreduction in H382S *At*64. The H382S mutant exhibited the typical spectral changes from $FAD_{ox}$ to $FADH^{-}$ upon white-light (> 430 nm) illumination with 5 mM of cysteine under anaerobic condition. Data between 580 and 620 nm are omitted due to an irreproducible bump in the region produced by instrumental problems.

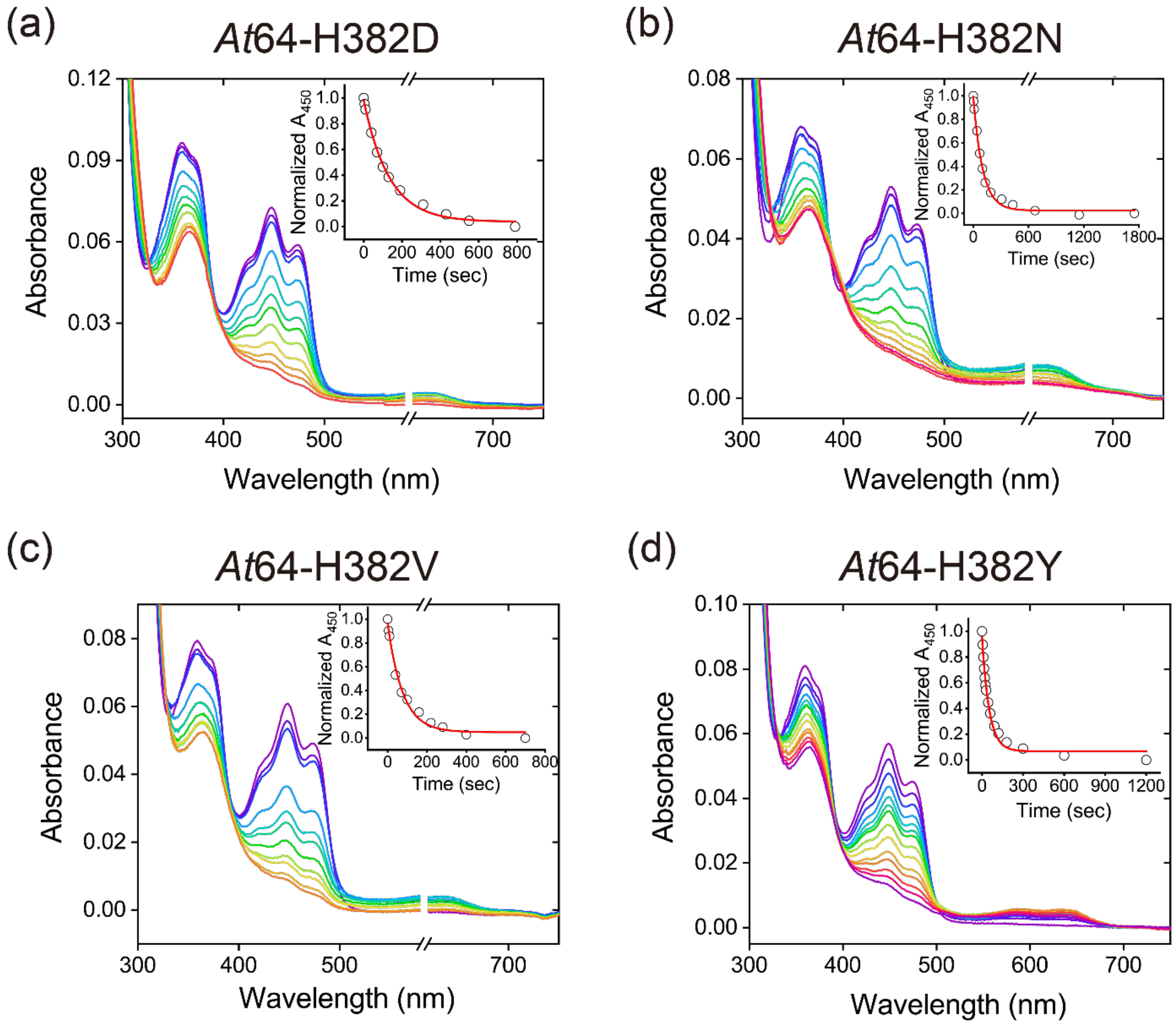


**Supplementary Fig. 2** Evolution of absorption spectra upon FAD photoreduction in the (a) H382D, (b) H382N, (c) H382V, and (d) H382Y mutants of *At*64. The plots of normalized $A_{450}$ against time are reasonably well fitted with monoexponential functions as shown in the insets. Data between 580 and 620 nm for (a), (b), and (c) are omitted due to an irreproducible bump in the region produced by instrumental problems.

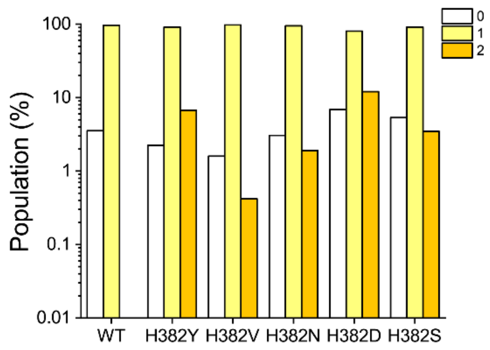


**Supplementary Fig. 3** Water molecules distribution in the first water shell, *i.e.*, within 3.4 Å of the nitrogen atom of the indole ring of Trp3H, for *At*64 variants. The numbers of water molecules within the shell in a frame are averaged over all the frames.

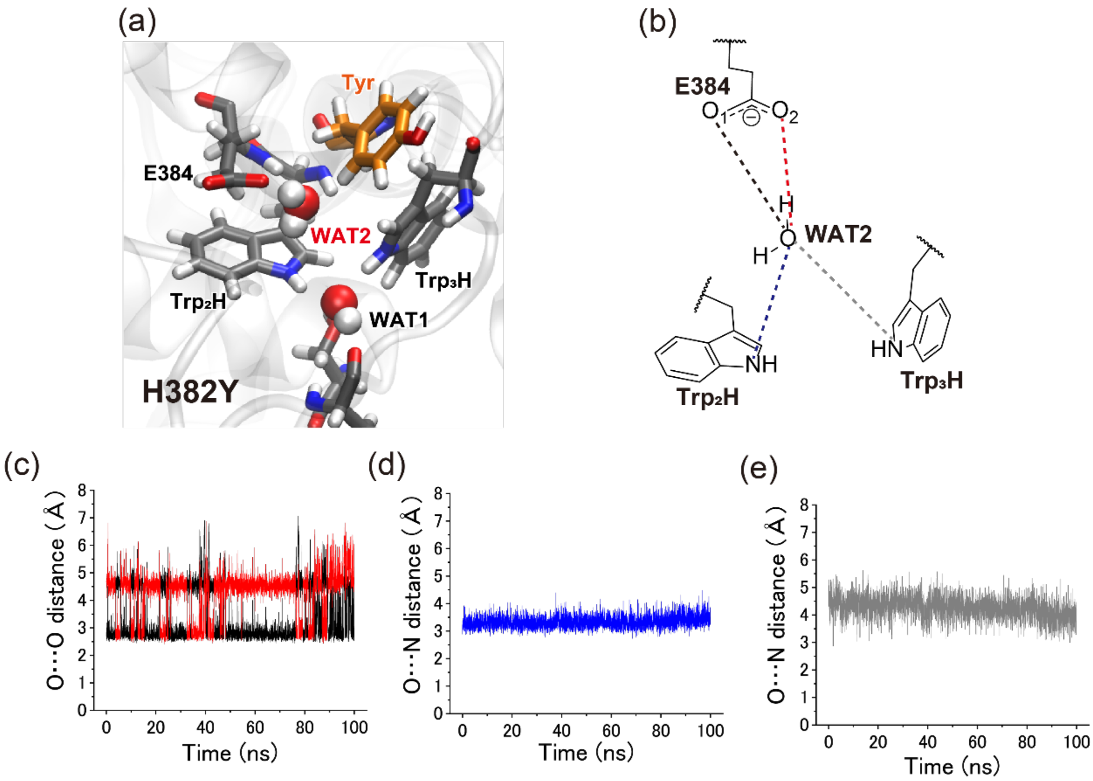


**Supplementary Fig. 4** Orientation of the stably captured water molecule (WAT2) in H382Y *At*64. (a) A snapshot from the MD simulation for H382Y. (b) A schematic view of WAT2 recognition site. (c) The time development of the distance between the $O_1$ / $O_2$ atoms of the E384 side chain and the O atom of WAT2 plotted in black / red. (d) The time development of the distance between the O atom of WAT2 and the N atom of $Trp_2H$ plotted in blue. (e) The time development of the distance between the O atom of WAT2 and the N atom of $Trp_3H$ plotted in gray.

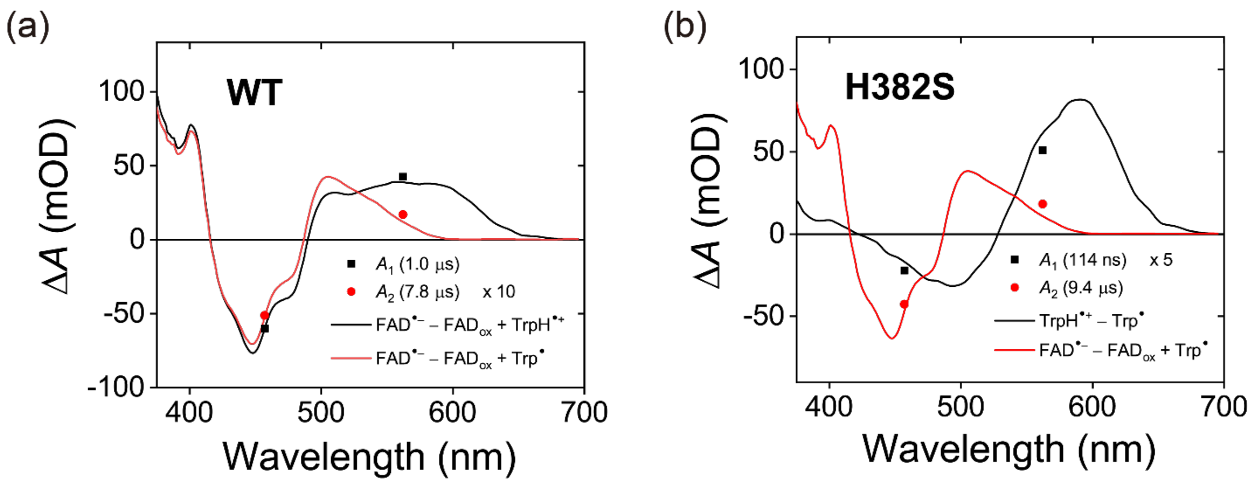


**Supplementary Fig. 5** Superimposition of the phase amplitudes obtained from the biexponential fits of the transient absorption changes for (a) WT and (b) H382S *At*64 (main text Fig. 6a and 6b, respectively) with the expected difference spectra (lines) reflecting the main underlying processes of the given kinetic phases (recombinations of the $FAD^{•–}$ $TrpH^{•+}$/$Trp^{•}$ radical pairs to $FAD_{ox}$ + TrpH (TrpH does not absorb in the shown region) and/or $TrpH^{•+}$ deprotonation to $Trp^{•}$).

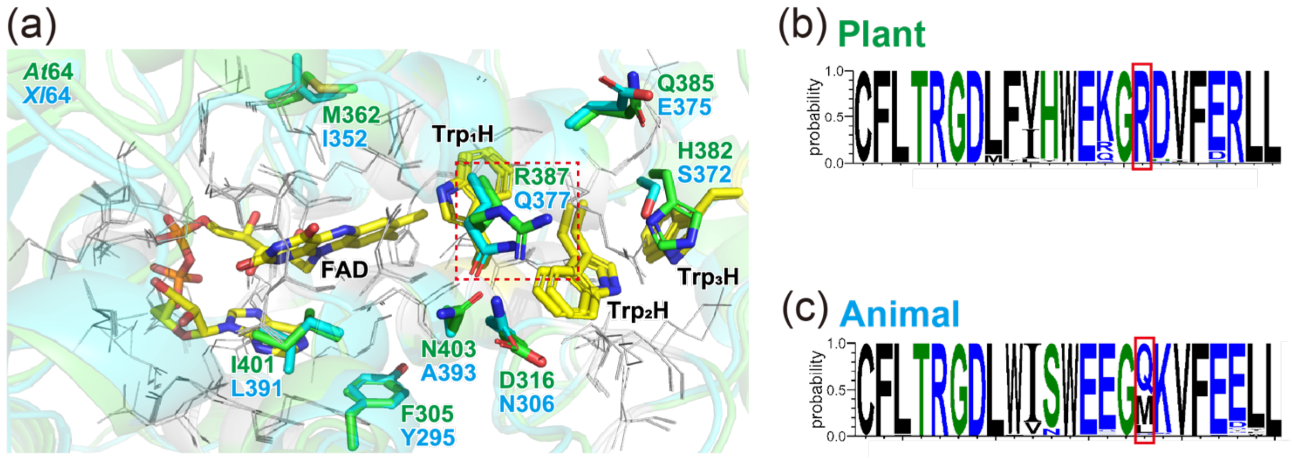


**Supplementary Fig. 6** Primary and tertiary structural analyses of the environment of the electron-transferring chain in plant and animal (6-4) PL orthologues. (a) The three-dimensional structures of *At*64 and *Xl*64 are superimposed. The residues within 4 Å of FAD, $Trp_1H$, and $Trp_2H$ are shown, and the white-grey colored residues in lines are conserved between *At*64 and *Xl*64. Residues unique to *At*64 and *Xl*64 are colored in green and cyan, respectively. Differences in the residues in the red dashed frame (R387 in *At*64 and Q377 in *Xl*64) could have an impact on the $FAD^{\bullet-} \cdots TrpH^{\bullet+}$ charge separation along the respective ET chains. (b and c) Primary structural analyses of (b) plant and (c) animal (6-4) PL orthologues. The residues in the red frame correspond to R387 in *At*64 and Q377 in *Xl*64.

**Supplementary Table 1.** Population of the frame, in which more than three water molecules were detected within 5.0 Å of the nitrogen atom of the indole ring of $Trp_3H$.

| | Population (%) |
|---|---|
| *At*64 (WT) | 1.42 |
| H382Y | 7.58 |
| H382V | 8.08 |
| H382N | 19.6 |
| H382D | 77.9 |
| H382S | 35.3 |

**Supplementary Table 2.** The distance between the oxygen atom of WAT1 and the nitrogen atom of the indole ring of $Trp_3H$ ($d_{O...N}{}^{1}$) during 100 ns simulation time for WT *At*64 and its H382 variants.

| | $d_{O...N}{}^{1}$ (Å) |
|---|---|
| *At*64 (WT) | 3.04 ± 0.19 |
| H382Y | 3.01 ± 0.17 |
| H382V | 2.98 ± 0.15 |
| H382N | 3.02 ± 0.17 |
| H382D | 3.10 ± 0.25 |
| H382S | 3.06 ± 0.20 |

**Supplementary Table 3.** Total number of different water molecules coming within 3.4 Å of the nitrogen atom of the indole ring of $Trp_3H$ over the 100 ns simulation.

| | Total number of water molecules |
|---|---|
| *At*64 (WT) | 1 |
| H382Y | 2 |
| H382V | 6 |
| H382N | 17 |
| H382D | 61 |
| H382S | 44 |

**Supplementary Table 4.** Calculated values of free energy difference ($\Delta G$) between the $Trp_2H^{\bullet+}$ state and the $Trp_3H^{\bullet+}$ state and reorganization energy ($\lambda$) followed by the electron transfer for WT and H382S.

| | $\Delta G$ (eV) | $\lambda$ (eV) |
|---|---|---|
| *At*64 (WT) | -0.577 | 1.582 |
| *At*64-H382S | -0.536 | 1.645 |

**Supplementary Table 5.** Primers for PCR amplification.

| Entry | Description | | | Sequence* |
|---|---|---|---|---|
| 1 | *At*64 | W329F | Forward | 5′-TGCAAACAGATTCCATTCAACGAGGATCAT-3′ |
| | | | Reverse | 5′-ATGATCCTCGTTGAATGGAATCTGTTTGCA-3′ |
| 2 | | H382D | Forward | 5′-GGATCTGTTCATAGATTGGGAACAAGGGCG-3′ |
| | | | Reverse | 5′-CGCCCTTGTTCCCAATCTATGAACAGATCC-3′ |
| 3 | | H382N | Forward | 5′-GGATCTGTTCATAAACTGGGAACAAGGGCG-3′ |
| | | | Reverse | 5′-CGCCCTTGTTCCCAGTTTATGAACAGATCC-3′ |
| 4 | | H382S | Forward | 5′-GGATCTGTTCATATCTTGGGAACAAGGGCG-3′ |
| | | | Reverse | 5′-CGCCCTTGTTCCCAAGATATGAACAGATCC-3′ |
| 5 | | H382V | Forward | 5′-GGATCTGTTCATAGTTTGGGAACAAGGGCG-3′ |
| | | | Reverse | 5′-CGCCCTTGTTCCCAAACTATGAACAGATCC-3′ |
| 6 | | H382Y | Forward 1 (with *Nde*I site) | 5′-CCGCGCGGCAGCCATA TGGCTACTGGATCCGGT-3′ |
| | | | Reverse 1 | 5′-CCTTGTTCCCAATATATGAACAGAT-3′ |
| | | | Forward 2 | 5′-ATCTGTTCATATATTGGGAACAAGG-3′ |
| | | | Reverse 2 (with *Xho*I site) | 5′-GTGGTGGTGCTCGAGCT ATTTGAGTTTTGGTCGTTG-3′ |
| 7 | *Xl*64 | W370F/ S372H | Forward 1 (with *Nde*I site) | 5′-CCGCGCGGCAGCCATAT GAGGCACAATTCCATCCA-3′ |
| | | | Reverse 1 | 5′-TTCTTCCCAATGTATGAAGAGGT-3′ |
| | | | Forward 2 | 5′-ACCTCTTCATACATTGGGAAGAA-3′ |
| | | | Reverse 2 (with *Xho*I site) | 5′-GTGGTGGTGCTCGAGTTAT TTTTTCTTGAACAATTCTGCC-3′ |
| 8 | Subcloning primer | | Forward (with *Nde*I site) | 5′-CCGCGCGGCAGCCATA TGGCTACTGGATCCGGT-3′ |
| | | | Reverse (with *Xho*I site) | 5′-GTGGTGGTGCTCGAGCT ATTTGAGTTTTGGTCGTTG-3′ |

*The codons generating desired mutations are underlined.

## Supplementary References


1. Altschul, S. F., Gish, W., Miller, W., Myers, E. W. & Lipman, D.J. Basic local alignment search tool. *J. Mol. Biol.* **215**, 403-410 (1990).
2. Papadopoulos, J. S. & Agarwala, R. COBALT: constraint-based alignment tool for multiple protein sequence. *Nucleic Acids Res.* **41**, W34-40 (2013).
3. Crooks, G. E., Hon, G., Chandonia, J. M. & Brenner, S. E. WebLogo: A sequence logo generator. *Genome Res.* **14**, 1188-1190, (2004).
4. Byrdin, M., Thiagarajan, V., Villette, S., Espagne, A. & Brettel, K. Use of ruthenium dyes for subnanosecond detector fidelity testing in real time transient absorption. *Rev. Sci. Instrum.* **80**, 043102 (2009).
5. Müller, P. & Brettel, K. $[Ru(bpy)_3]^{2+}$ as a reference in transient absorption spectroscopy: differential absorption coefficients for formation of the long-lived $^3$MLCT excited state. *Photochem. Photobiol. Sci.* **11**, 632-636 (2012).
6. Lachish, U., Infelta, P. P. & Grätzel, M. Optical absorption spectrum of excited ruthenium tris-bipyridyl ($Ru(bpy)_3^{2+}$). *Chem. Phys. Lett.* **62**, 317-319 (1979).
7. Case, D. A. *et al.* Amber 2016. University of California, San Francisco.
8. Hosokawa, Y., Sato, R., Iwai, S. & Yamamoto, J. Implications of a water molecule for photoactivation of plant (6–4) photolyase. *J. Phys. Chem. B* **123**, 5059-5068 (2019).
9. Waterhouse, A. *et al.* SWISS-MODEL: Homology Modelling of Protein Structures and Complexes. *Nucleic Acids Res.* **46**, W296−W303 (2018).
10. Maier *et al.* ff14SB: Improving the Accuracy of Protein Side Chain and Backbone Parameters from ff99SB. *J. Chem. Theory Comput.* **11**, 3696−3713 (2015).
11. Jorgensen, W. L., Chandrasekhar, J., Madura, J. D., Impey, R. W. & Klein, M. L. Comparison of Simple Potential Functions for Simulating Liquid Water. *J. Chem. Phys.* **79**, 926-935 (1983).
12. Ryckaert, J.-P., Ciccotti, G. & Berendsen, H. J. C. Numerical Integration of the Cartesian Equations of Motion of a System with Constraints: Molecular Dynamics of n-Alkanes. *J. Comput. Phys.* **23,** 327-341 (1977).
13. Darden, T., York, D. & Pedersen, L. Particle Mesh Ewald: An N·log(N) Method for Ewald Sums in Large Systems. *J. Chem. Phys.* **98**, 10089-10092 (1993).
14. Roe, D. R. & Cheatham, T. E. III. PTRAJ and CPPTRAJ: Software for Processing and Analysis of Molecular Dynamics Trajectory Data. *J. Chem. Theory Comput.* **9**, 3084-3095 (2013).
15. Case, D. A. *et al.* AMBER 2018. University of California, San Francisco.
16. Wang, J., Wolf, R. M., Caldwell, J. W., Kollamn, P. A. & Case, D. A. Development and testing of a general Amber force field. *J. Comput. Chem.* **25**, 1157-1174 (2004).
17. Singh, U. C. & Kollman, P. A. An Approach to Computing Electrostatic Charges for Molecules. *J. Comput. Chem.* **5**, 129-145 (1984).
18. Frisch, M. J. *et al.* Gaussian 16 Revision B01 Gaussian, Inc., Wallingford, CT, 2016.
19. Salomon-Ferrer, R. Goetz, A. W., Poole, D., Le Grand, S. & Walker, R. C. Routine microsecond molecular dynamics simulations with AMBER - Part II: Particle Mesh Ewald. *J. Chem. Theory Comput.* **9**, 3878-3888 (2013).
20. King, G. & Warshel A. Investigation of the free energy functions for electron transfer reactions. *J. Chem. Phys.* **93**, 8682 (1990).

21. de la Lande, A., Gillet, N., Chen, S. & Salahub, D. R. Progress and challenges in simulating and understanding electron transfer in proteins. *Arch. Biochem. Biophys.* **582**, 28－41 (2015).

22. Cailliez, F., Müller, P., Firmino, T., Pernot, P. & de la Lande, A. Energetics of photoinduced charge migration within the tryptophan tetrad of an animal (6–4) photolyase. *J. Am. Chem. Soc.* **138**, 1904－1915 (2016).
